# Monitoring Differential Subsidence along the Beijing–Tianjin Intercity Railway with Multiband SAR Data

**DOI:** 10.3390/ijerph16224453

**Published:** 2019-11-13

**Authors:** Min Shi, Beibei Chen, Huili Gong, Xiaojuan Li, Wenfeng Chen, Mingliang Gao, Chaofan Zhou, Kunchao Lei

**Affiliations:** 1 Key Laboratory of Mechanism, Prevention and Mitigation of Land Subsidence, MOE, Capital Normal University, Beijing 100048, ChinaB312@cnu.edu.cn (M.G.); B328@cnu.edu.cn (C.Z.); 2 Beijing Laboratory of Water Resources Security, Capital Normal University, Beijing 100048, China; 3 Base of the State Key Laboratory of Urban Environmental Process and Digital Modeling, Capital Normal University, Beijing 100048, China; 4 Key Laboratory of Tibetan Environmental Changes and Land Surface Processes, Institute of Tibetan Plateau Research, Chinese Academy of Sciences (CAS), Beijing 100101, China; 5 Beijing Institute of Hydrogeology and Engineering Geology, Beijing 100195, China; leikunchao123@126.com

**Keywords:** high-speed railway, subsidence, different settlement, InSAR

## Abstract

High-speed railways have strict standards of infrastructure deformation and post-construction settlement. The interferometric synthetic aperture radar (InSAR) has the ability to detect ground deformation with a high accuracy and wide coverage and is becoming a useful tool for monitoring railway health. In this study, we analyzed the Beijing–Tianjin Intercity Railway (BTIR) track using InSAR time-series analysis with different data sets. First, by using RADARSAT-2 images, we examined the areas along the BTIR with significant subsidence. Then, we characterized these areas by means of X-band TerraSAR-X data. We adopted the expectation (Ex) and entropy (En) method, combined with GIS spatial analysis, to analyze the ground settlement differences on both sides of the railway. The results show that the area with the most severe differential settlement occurs between 12 and 20 km along the railway and within 120 to 20 m on both sides of the Chaoyang–Tongzhou section (CTS). Thereafter, we analyzed the reasons for the large difference in this area by considering different factors, e.g., regional land subsidence, groundwater level changes, and the dynamic load. In addition, we studied the impact of regional subsidence on the safe operation of the BTIR. The results show that the maximum different settlement along the BTIR is within the safe range, according to the high-speed railway design standard between 2010 and 2015. This study aims to provide technical support for assessing the impact of subsidence on the safety of railway operations.

## 1. Introduction

As a common widespread geological phenomenon, land subsidence has irreversible and cumulative characteristics. The spatial and temporal development of land subsidence affects the structure and stability of buildings and urban infrastructure, even causing financial losses and fatalities. With the availability of a short revisit time and high-resolution synthetic aperture radar (SAR) satellites and multi-temporal interferometric synthetic aperture radar (MT-InSAR) techniques, many studies [1] have focused on using the interferometric synthetic aperture radar (InSAR) ground deformation monitoring of buildings [2,3,4,5,6,7,8], airports [9,10,11], large man-made linear features [12,13,14,15,16,17], etc. 

Large man-made linear features play an important role in regional economic and social development. Regional land subsidence has negative effects on the slope and stability of large man-made linear features. High-speed railways are linear features with large extents and irregular distributions. Traditional deformation observation depends on set leveling and GPS on both sides of the line to form the settlement monitoring network. However, for railways, traditional methods have the disadvantage of a low spatial sampling density, long observation period, and high cost. InSAR, as a powerful land deformation mapping tool, overcomes these limitations. MT-InSAR techniques, such as Persistent Scatterers InSAR (PSI) [18] and the Small Baseline Subset (SBAS) approach [19], have been useful for detecting time-dependent subsidence with a high precision. With the launch of high-resolution SAR satellites, the MT-InSAR technique has been successfully used to study long-term micro-deformation along linear features under all terrain conditions and at large spatial scales. 

The Beijing–Tianjin Intercity Railway (BTIR) is located in the northern part of the North China Plain (NCP), where covers the largest subsidence area on the planet. The total length of the railway is 120 km, and most of the tracks are ballastless tracks, which have strict subgrade settlement requirements, especially uneven settlement. To ensure the safety of the BTIR, it is necessary to monitor the structural health of the railway throughout its whole life cycle. With the InSAR technique, it is possible to capture the settlement section along the railway and continuously monitor it, providing technical support for railway operation safety assessment. Previous studies have mainly focused on using the time-series InSAR approach to monitor the subsidence along the railway [20,21,22]. Combined with groundwater data, geological data were used to analyze the development of land subsidence. In this paper, we monitor the subsidence along the railway by using SAR data with different resolutions. We combine GIS spatial analysis with the entropy (En) method to obtain the influence range of differential settlement in the horizontal and vertical directions of the railway. 

This article is organized as follows. The study site and SAR data are described in Section 2. The Stanford Method for Persistent Scatter (StaMPS) technique and the Ex and En method used in this study are presented in Section 3. In Section 4, we use RADARSAT-2 images to estimate the time-series deformation along the BTIR. X-band images with a 3 m resolution were used to examine the key impact area of land subsidence along the railway. With the InSAR results, the spatial distribution and time-series development characteristics of subsidence along the railway are analyzed by the En method. Finally, serious differential settlement zones on both sides of the railway are detected. Discussions and conclusions of this study are provided in Section 5 and Section 6, respectively.

## 2. Study Area and Data Description

The BTIR is located in the northern part of the NCP and is a platform used to boost the formation and development of Beijing and Tianjin, which was built in 2005 and began to operate in 2008, with a maximum speed of 350 km/h. The BTIR crosses or passes close to major subsidence zones in the NCP. Subsidence is one of the most important geological natural hazards in the NCP, especially in Beijing and Tianjin. From 1935 to 2013, the maximum accumulated subsidence grew to 1.49 m, and the area of accumulated settlement over 50 mm in the Beijing Plain reached more than 4300 km^2^ [23]. In Tianjin, the average subsidence was estimated to be 26 mm/yr, and the maximum subsidence reached 117 mm/yr, in 2015, exhibiting an attenuation trend [24,25,26]. The area with a subsidence rate higher than 10 mm was 7701 km^2^. Ballastless tracking, which is used in the BTIR, has strict subgrade deformation and post-construction settlement requirements. Long-term land subsidence threatens the stability of the railway. This condition not only results in higher maintenance costs, but also threatens the safety of passengers. Therefore, the BTIR experiences higher potential risks of subsidence to passenger safety than traditional railways due to its high-speed operation and frequency schedule. It is very important to define the rates and spatial patterns of land subsidence along the railway.

**Figure 1:**
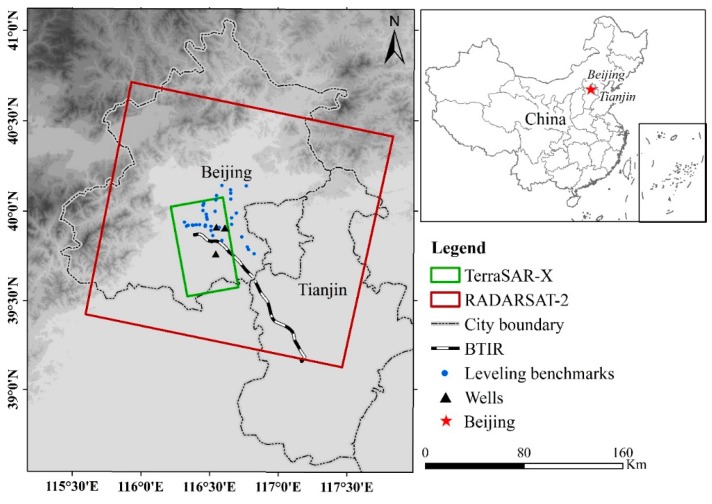
The location of the Beijing–Tianjin Intercity Railway. The boxes represent the frames of the synthetic aperture radar (SAR) data used. The blue circles indicate the geographical locations of the benchmarks of the ground leveling survey. The black triangles indicate the monitoring points of the groundwater level.

In this study, we used two stacks of SAR data to monitor the subsidence along the BTIR, C-band RADARSAT-2, and X-band TerraSAR-X/TanDEM-X (TSX/TDX) data. The coverage of the two-band data is shown in Figure 1. RADARSAT-2 is an Earth observation satellite launched by the Canadian Space Agency (CSA) on 14 December 2007, with a revisit frequency of 24 days and orbit altitude of 798 km. The SAR image stack covers an area of approximately 22,500 km^2^ (the blue box in Figure 1), thus basically covering the whole BTIR. TSX/TDX is managed by the German Aerospace Center and EADS Astrium. The TSX, launched on 15 June 2007, carries a steerable X-band SAR sensor with an orbit altitude of 514 km. As the twin satellite of the TSX, the TDX satellite (with a TSX add-on for digital elevation measurements) is almost identical to the TSX, which was launched in 2010.

## 3. Method

### 3.1. StaMPS Measurement

**Figure 2:**
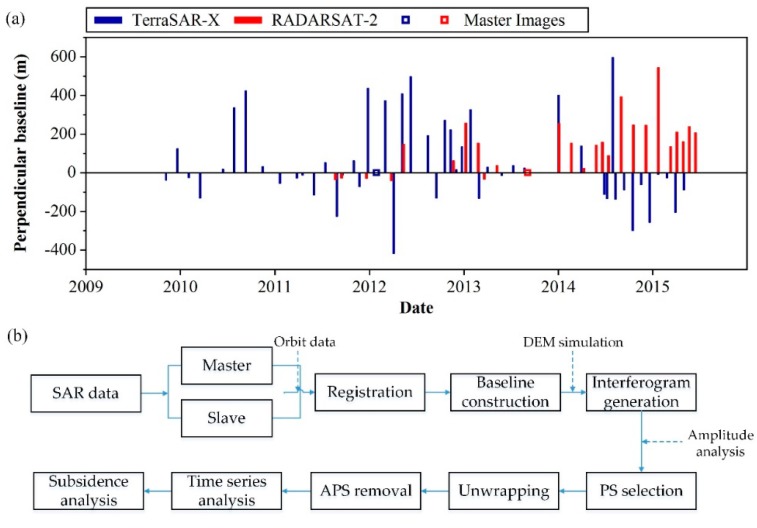
(**a**) Temporal-spatial baseline distributions of the TerraSAR-X and RADARSAT-2 image stacks in this study. (**b**) The framework of the StaMPS technique. The interferometric synthetic aperture radar (InSAR) is sensitive to the line-of-sight (LOS) direction, while leveling measurement is sensitive along the vertical direction. Previous studies have shown that the Beijing Plain presents a low relative horizontal movement of 1.57–1.93 mm/yr [29,30]. Therefore, we assumed that the detected movements were mostly in the vertical direction during the study period in this research. Then, the vertical deformation could be derived from the following equation and validated with ground leveling data.

StaMPS was developed by Hopper et al., and is applicable to low-amplitude natural targets and requires no prior deformation model for identifying and processing persistent scatterer (PS) [27,28]. In this study, we applied StaMPS to monitor the subsidence along the railway in two time periods with SAR data. Twenty-six RADARSAT-2 images in the Stripmap mode from the ascending track with vertical-vertical (VV)polarization were used to monitor the subsidence between January 2012 and November 2015. The resolution of the SAR data is 30 m, and details of the perpendicular baseline are presented in Figure 2a. Forty-nine TSX/TDX SAR images acquired between April 2010 and October 2015 from the ascending track with the InSAR method were used to monitor subsidence along the railway in Beijing. All the TSX/TDX images were obtained in the Stripmap mode with horizontal-horizontal (HH) polarization and with a resolution of up to 3 m. The temporal-spatial baselines of these X-band images are shown in Figure 2a with a blue color. The main procedures of the StaMPS technique are as follows: interferogram formation, phase stability estimation, PS selection, and displacement estimation. The Digital Elevation Model (DEM) from the Shuttle Radar Topography Mission (SRTM), with a 90 m resolution was used to remove the topographic phase.

(1) $$d_{v}=d_{LOS}/\cos\theta ,$$

where $d_{v}$ is the vertical deformation, $d_{LOS}$ is the line-of-sight (LOS) direction deformation, and θ is the SAR incidence angle.

### 3.2. Expectation and Entropy Method

Ex and En represent values that are fully compatible with the qualitative concept and the uncertainty of the concept in the cloud model, respectively [31,32]. The values of Ex and En can be calculated as follows [33]:

(2) $$Ex=\bar{X},$$

where $\bar{X}=\left(1/N\right)\sum_{i=1}^{N}x_{i}$, and $x_{i}$ is the settlement value of the PS points in the test area.

Then, En can be calculated by Equation (3):

(3) $$En=\sqrt{\pi /2}\times \left(1/N\right)\sum_{i=1}^{N}\left|x_{i}-Ex\right|\;.$$

In a previous study, researchers used Ex and En to characterize the difference of the settlement along the subway [34]. In this paper, Ex and En are used to represent the overall level and the nonuniformity degree of land subsidence, respectively. The spatial and temporal En method was applied to capture the boundary affected by subsidence along the railway. Considering the BTIR as a cyclic dynamic load, the effect of the dynamic load on land subsidence was analyzed. First, we created a ring buffer at regular intervals on both sides of the railway. Then, Ex and En values of all PS points in the buffer were calculated to determine the overall level and spatial nonuniformity degree of land subsidence, respectively. Finally, the changes in Ex and En were compared to determine the severe difference subsidence area. The temporal characteristics of subsidence in this area were examined by calculating the time-series values of Ex and En.

## 4. Results

The InSAR measurement results are examined in this section. We generated two interferometric datasets for RADARSAT-2 (January 2012–November 2015) and TSX (April 2010–October 2015) using the method described in Section 3.1. Then, validation was performed using ground leveling data. 

### 4.1. InSAR Measurements

**Figure 3:**
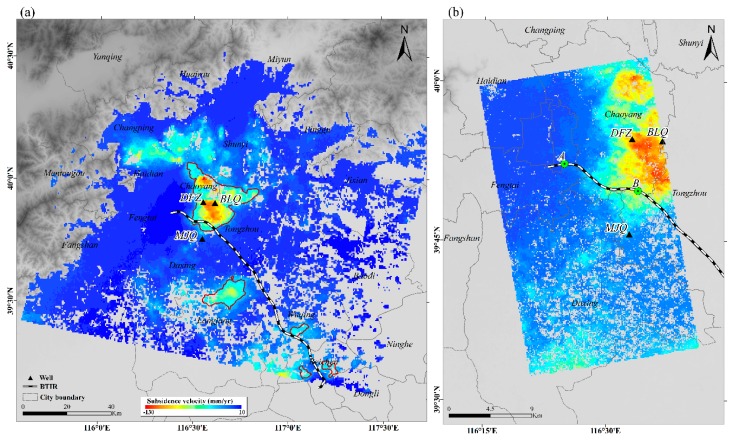
Line-of-sight velocity map and InSAR measurement profile along the Beijing–Tianjin Intercity Railway (BTIR): (**a**) RADARSAT-2 ascending track (January 2012–November 2015) with a maximum subsidence of 130 mm/yr and maximum uplift of 10 mm/yr. (**b**) TSX/TDX ascending track (April 2010–October 2015) with a maximum subsidence of 121 mm/yr and a minimum value of 4 mm/yr.

Figure 3a shows the average surface deformation map from January 2012 to November 2015 measured by RADARSAT-2. Negative values indicate subsidence, and positive values represent uplift. During the monitoring period, significant land subsidence occurred in the Beijing Plain, especially in the Changping, Shunyi, Tongzhou, and Chaoyang districts, with the maximum settlement rate reaching more than −100 mm/yr. We used the 30 mm/yr deformation line as a boundary of the subsidence regions, and the results are shown in Figure 3a. The BTIR crosses the edges of the Chaoyang–Tongzhou and Wuqing funnels, which have maximum subsidence rates of −130 and −44 mm/yr, respectively. The observed maximum subsidence rate reached −71 mm/yr for the Langfang funnel, and the BTIR was approximately 7 km from the edge of the center of the subsidence area. In addition, the distance between the Beichen subsidence zones and the BTIR is 2 and 4 km.

**Figure 4:**
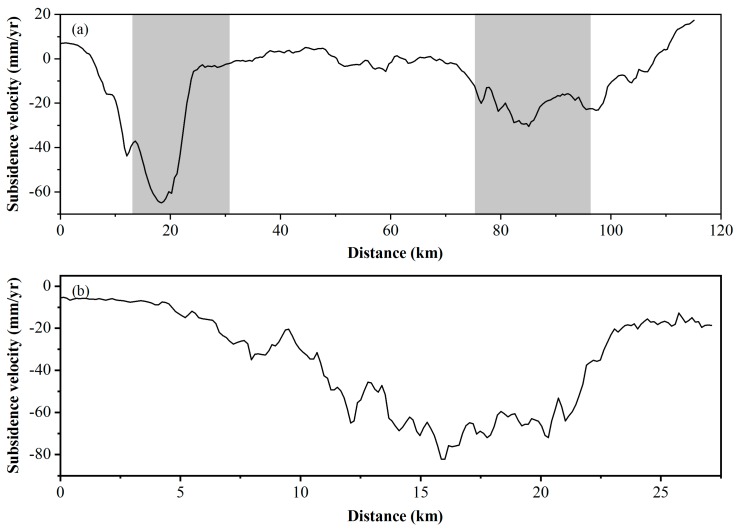
(**a**) Profile of RADARSAT-2 displacement (2012–2015) along the BTIR. Two major subsidence zones could be identified along the entire distance and are marked with gray shadows. (**b**) Profile of TerraSAR-X (TSX) displacement along the BTIR (2010–2015).

We plotted the deformation profile along the BTIR. As shown in Figure 4, the profile along the BTIR exhibits a significant difference along the railway. The displacement rate along the railway sharply transitions between 5–26 km and 78–192 km. Combined with Figure 3a, these two sections correspond to the Chaoyang–Tongzhou section (CTS) and Wuqing–Beichen section (WBC) of the railway, respectively. As a result, two significant settlement sections of the BTIR have been identified.

RADARSAT-2 is a C-band satellite with a larger coverage than the X-band satellite. Through RADARSAT-2 data, displacement information of the entire BTIR has been obtained and the sinking area along the railway could be detected. TSX SAR data can help us focus the attention on the sinking area along the railway with a higher resolution and shorter revisit time. We identified a serious settlement zone on the northeast side of the CTS. The settlement along the CTS is quite different, with relatively small subsidence values (<20 mm/yr) between 0–6 km and 23–27 km, and relatively large values (>20 mm/yr) in the middle section along the railway (in Figure 4b).

### 4.2. Comparison with Leveling Measurements

To evaluate the accuracy of the StaMPS measurements, leveling survey data were used for precision validation (indicated by the blue circles in Figure 1). In our study, ground leveling survey data were acquired annually from September 2012 to September 2013. Then, each benchmark was used as the center, and the average displacement of pixels with a certain radius (100 m in our case) was obtained as the corresponding InSAR measurement. 

**Figure 5:**
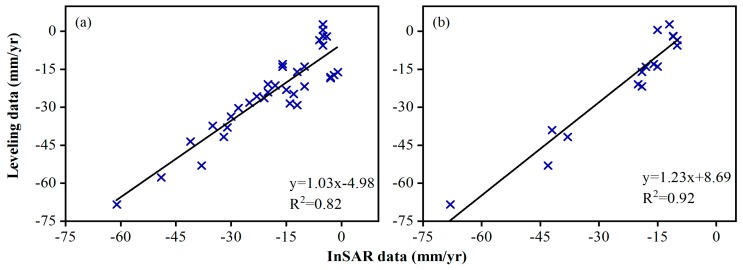
Comparison of the InSAR and ground leveling velocities from 2012 to 2013. (**a**) Regression analysis of the average velocity between the RADARSAT-2 results and leveling measurements. (**b**) Regression analysis of the average velocity between the TSX/TanDEM-X (TDX) results and leveling measurements.

Figure 5a shows a scatter plot generated by RADARSAT-2 InSAR measurements and ground leveling surveys at the 34 benchmarks. A quantitative comparison of the average subsidence velocity between these two measurement techniques is listed in Table 1. The maximum difference between the two measurements is 17.2 mm/yr, and the minimum error is 0.6 mm/yr, with a mean difference of 7.0 mm/yr. The correlation coefficient (R^2^) is 0.82.

The InSAR analysis average subsidence rate of the TSX was validated with leveling survey data from 16 benchmarks. Table 2 shows the differences between the two measurements, with a maximum difference of 15.6 mm/yr and a minimum difference of 0.4 mm/yr. The linear regressions between the PS points and leveling points are shown in Figure 5b. The correlation coefficient (R^2^) of 0.92 indicates a good agreement between these two methods.

### 4.3. Temporal-Spatial Evolution of Land Subsidence along the Railway by the Ex and En Method

As mentioned in Section 4.1, the subsidence velocity along the BTIR exhibits a significant difference, as indicated by RADARSAT-2 InSAR analysis. Two railway sections with severe subsidence fluctuations were identified: the CTS and the WBC. Then, X-band data were used to obtain the temporal evolution of land displacement along the CTS.

**Figure 6:**
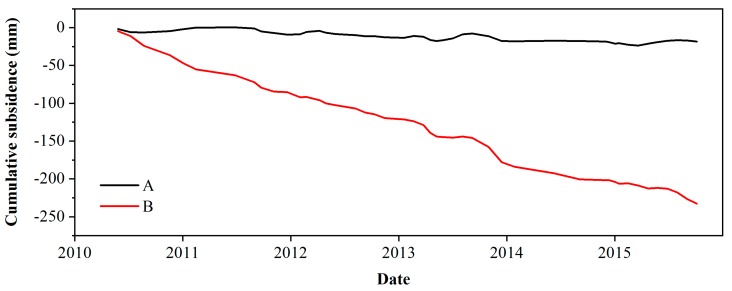
Subsidence history of points along the railway. The locations of points are shown in Figure 3b.

To study the track settlement in the different regions, the InSAR measurements at two representative points were further analyzed, including one point far from the settlement funnel and another point close to the funnel. The settlement histories of the points are shown in Figure 6. The settlement of point A remains stable from 2010 to 2015, while point B sinks faster than point A. This phenomenon proves that the increase in settlement varies at different locations along the railway, and it is necessary to identify the section with severe settlement.

**Figure 7:**
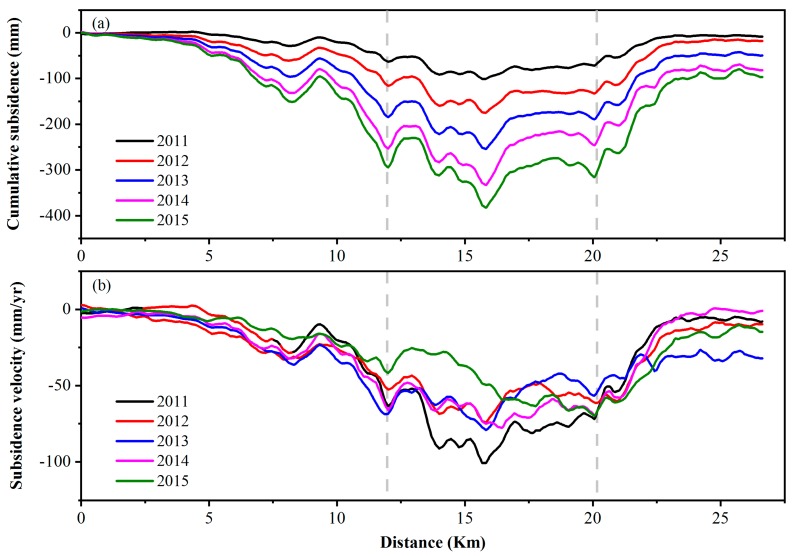
Time-series cumulative subsidence (**a**) and subsidence velocity (**b**) along the Chaoyang–Tongzhou section from April 2010 to October 2015. The significant subsidence zone is marked by a gray dashed line and its location is from 12 to 20 km.

We plotted deformation profiles over time for the railway, and the results are shown in Figure 7. The cumulative settlement of the railway has increased over time, as shown in Figure 7a. The region settles more seriously along the BTIR between 12 and 20 km than in other sections, with the maximum cumulative settlement reaching 400 mm in 2015. Figure 7b illustrates the average subsidence velocity, with major fluctuations in the CTS section. The maximum and minimum settlement rates appear in 2011 and 2015 and are −77 and −47 mm/yr, respectively. In the whole CTS, the settlement is more serious from 12 to 20 km, while the settlement velocity slows down from 2011 to 2015.

**Figure 8:**
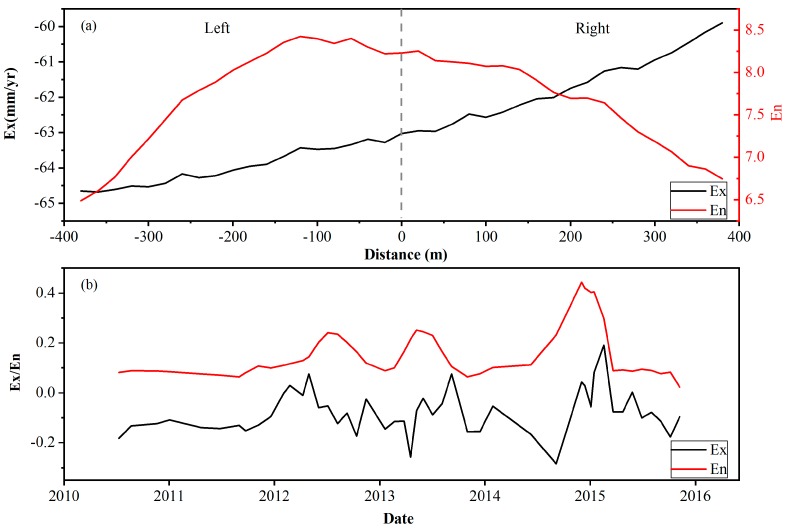
(**a**) Expectation (Ex) and entropy (En) values in each buffer zone from 12 to 20 km. (**b**) Ex and En value time series in the zone with a significant nonuniform settlement (April 2010–October 2015).

For the key subsidence area determined above, 20 buffer zones were established on both sides of the railway, with a radius of 20 m. Then, 10,000 points were randomly generated in the buffer zones to extract the corresponding settlement values. Finally, the expectation and entropy values of all points in each buffer zone were calculated. The results are shown in Figure 8a. Based on the direction of Beijing to Tianjin, the left side of the railway is negative and the right side is positive. In the range of 120 m on the left side of the line, the value of En increases with decreasing Ex. Outside the buffer zones, the overall value of the two parameters shows a downward trend. The Ex and En values increase synchronously within 20 m on the right side of the railway, and the En value reaches a maximum at 20 m. The value of Ex in the other areas shows a trend of continuous increase, and the En value shows the opposite trend. In this paper, the Ex and En values represent the overall level of deformation and the dispersion degree of deformation, respectively. From these values, it can be ascertained that the nonuniform settlement degree along the line is relatively high in the range of 120–20 m from north to south.

Then, the evolution characteristics of the uneven subsidence were examined by time-series entropy analysis of the railway line from 12 to 20 km and from 120 m in the north to 20 m in the south. As shown in Figure 8b, in terms of the time series, the change trends of the expectation and entropy have a good consistency. The values of Ex and En fluctuate up and down in the different time periods, with a large fluctuation between 2012 and 2014. This finding means that the regional settlement rate generally increases during this period, and the degree of nonuniformity is high. The possible trigger factors for this phenomenon will described in Section 5.1.

## 5. Discussion

### 5.1. Subsidence Factors along the Railway

**Figure 9:**
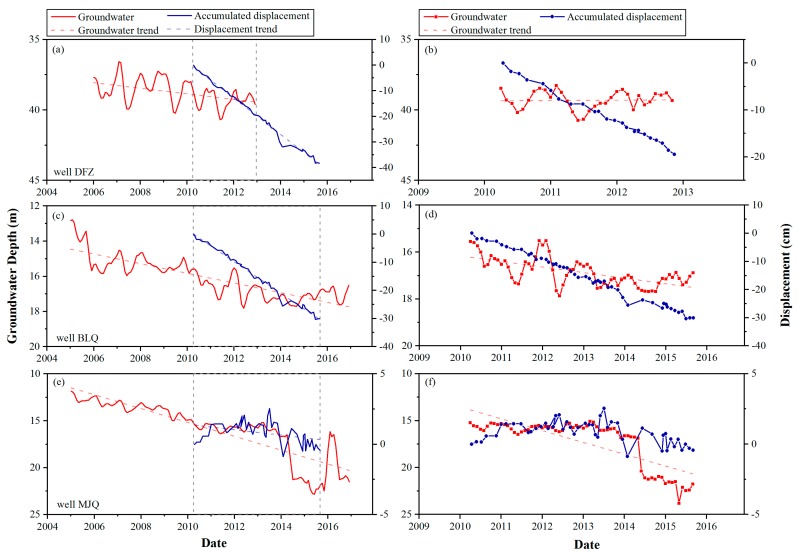
Comparison of the well-logging groundwater depth and InSAR time-series measured displacement between 2005 and 2017. The groundwater change trend is indicated by the dashed line. (**a**,**c**,**e**) shows a comparison of long term groundwater variations with InSAR displacement time series. (**b**,**d**,**f**) shows a close-up view of the water level trend in the time period covered by the InSAR results.

**Figure 10:**
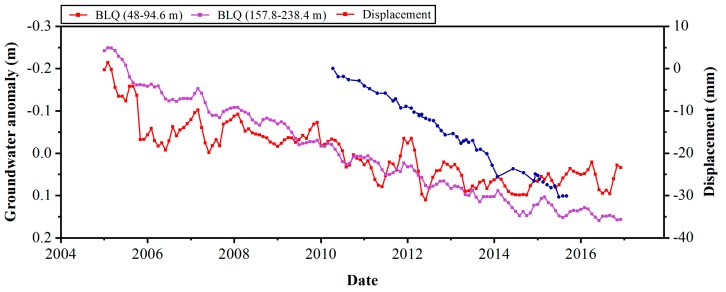
Comparison of well BLQ groundwater depth and InSAR measured displacement (2005–2016). Time series groundwater depth changes observed by well BLQ after the anomaly.

In Figure 9, the displacement time series from the TSX dataset is compared with groundwater data at three locations. The left panel shows a comparison of long term groundwater variations with InSAR displacement time series (in Figure 9a,c,e). The right panel shows a close-up view of the water level trend in the time period covered by the InSAR results (in Figure 9b,d,f). The details of wells are shown in Table 3. The groundwater data used in Figure 9 was monitored to a depth of less than 100 m. Wells Dengfuzhuang (DFZ) and Baliqiaocun (BLQ) are located near the center of the Chaoyang–Tongzhou funnel, while well Majuqiao (MJQ) is located far from the subsidence zone in Figure 3. We collected groundwater data for wells BLQ and MJQ from 2005 to 2016 and for well DFZ, only from 2006 to 2012. Periods of rapid and gentle declines, slight recoveries, and seasonal fluctuations of the groundwater level at the different locations can be observed in Figure 9a,c,e. The groundwater trend line (indicated by the red dashed line) changes in the three wells show the water level decline during the observation period. Due to the different variations in groundwater level at the different locations, the degree of subsidence is also different. With the long-term groundwater decline, the subsidence shows a continuous trend in well BLQ. However, there is still some inconsistency between the groundwater and surface displacement levels. At well BLQ, we collected groundwater depth data for different monitoring deeps. The comparison of InSAR displacement and two sets of groundwater depth data of well BLQ is shown in Figure 10. The results show that the groundwater change with monitoring deep under 100 m has a great impact on the development of surface deformation. This observation indicates that different depths of groundwater change contribute differently to land subsidence. 

A close-up view of the groundwater changes and trend lines for the time period of the InSAR measurements is shown in Figure 9b,d,f. The groundwater behavior differs at different locations. In wells DFZ and BLQ, we can observe clear seasonal fluctuation in the groundwater level. In well MJQ, the groundwater level was relatively stable before 2014, and there was a significant decline thereafter. A comparison of the groundwater levels with the InSAR displacement time series shows various relationships between the groundwater level and surface displacement. In well DFZ, the groundwater depth is stable, but land subsidence is significant. In well BLQ, ground subsidence coincides with a drop in the groundwater level. The observed changes in settlement slightly lag behind the groundwater level changes. In well MJQ, land subsidence is stable, and the changes in settlement slightly lag behind the groundwater level changes between 2010 and 2014. Then, the groundwater level decreased by nearly 6 m from 2014 to 2015, whereas land subsidence was stable. During the period of InSAR measurements, the various groundwater level changes induce different subsidence characteristics. 

**Figure 11:**
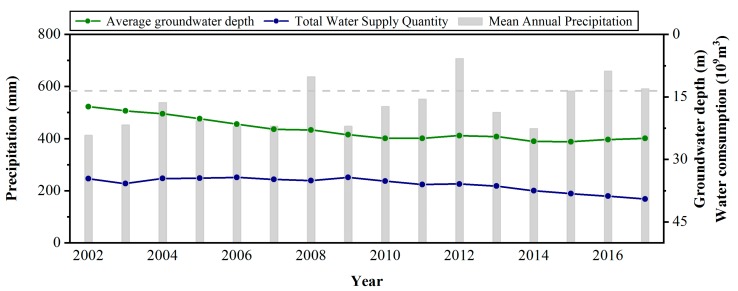
Annual precipitation, annual mean groundwater depth, and annual water consumption from 2002 to 2017. The average annual precipitation of 585 mm is indicated by the gray dashed line.

**Figure 12:**
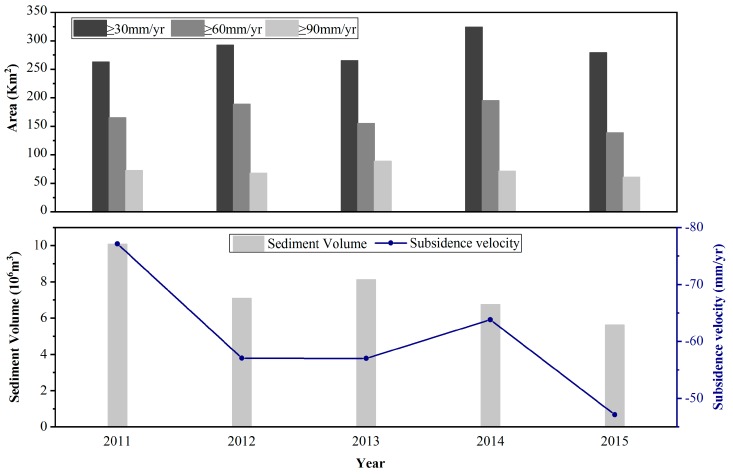
The upper panel is a plot of the annual deformation exceeding 30, 60, and 90 mm from 2011 to 2015. The lower panel is a plot of the annual volume and maximum rate of land subsidence in key areas from 2011 to 2015.

Previous research has revealed that the overexploitation of water resources is the main driving force for subsidence in the Beijing Plain [35,36]. Figure 11 shows the historical Beijing water resource information. From 2002 to 2011, the groundwater depth in the Beijing Plain continued to increase. This result means that the groundwater level continued to decline during this period. After 2014, the groundwater depth declined, despite the increase in total water consumption. This phenomenon may be because, after 2014, the annual precipitation was higher than the average annual rainfall, which increased the recharge. The inflow of water from the south has changed the water supply pattern in Beijing. A previous study showed that the area and volume of land subsidence in the Beijing Plain presented simultaneous decreasing trends [37]. We calculated the areas of the subsidence funnel exceeding 30, 60, and 90 mm from 2011 to 2015 based on the TSX results (Figure 12). Compared with 2011, the area with a settlement rate higher than 30 mm in 2015 is larger. However, areas with settlement rates higher than 60 and 90 mm are smaller than those in 2011. The change trend of the regional settlement area is basically consistent with the change in groundwater depth. With the subsidence development of the Chaoyang–Tongzhou funnel, the volume and subsidence velocity of the significant nonuniform settlement zone generally decrease from 2011 to 2015 (Figure 12). From 2011 to 2012, the volume of the significant nonuniform settlement zone decreased from 10.07 × 10^6^ to 7.08 × 10^6^ m^3^. Then, the volume of land subsidence presented increasing trends from 2012 to 2013. After 2013, the volume of land subsidence decreased from 8.12 × 10^6^ to 5.62 × 10^6^ m^3^. The maximum and minimum settlement rates were observed in 2011 and 2015 and were −77 and −47 mm/yr, respectively.

In this study, we used the space-time entropy to characterize the evolutionary characteristics of settlement along the high-speed railway and identify the key monitoring area of the railway. We regarded the high-speed rail as a cyclic dynamic load and analyzed the effect of rail operations on settlement, combined with the space-time entropy. According to Figure 8a, the entropy value is larger in the middle of the railway and smaller on both sides. This result indicates that railway operations may have some influence on the uneven regional settlement. The values of Ex and En display slight fluctuation between 2010 and 2011, as shown in Figure 8b. Examining the water resource information of Beijing, we found that the groundwater depth was stable between 2010 and 2011. Data from the three monitoring wells near the railway show a stable trend of the groundwater depth at the same time. Above all, we can infer that the dynamic load affects the development of nonuniform regional settlement.

### 5.2. Potential Risk Caused by Subsidence

The main impact of regional settlement on the BTIR is a change in the slope of the line. According to the standards of high-speed railways in China, the maximum slope change in the BTIR design for 100 years is 20‰. The calculation equation of the differential settlement slope between two points is

(4) i=Δh/L=n(b−a)/L

where Δh is the settlement difference between two points, L is the distance of the line between the two points, n is the settlement calculation period, and a and b are the settlement rates of the two points.

**Figure 13:**
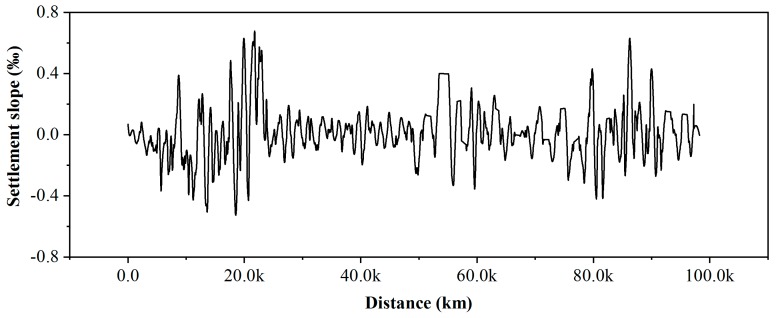
Settlement slope along the railway obtained from the RADARSAT-2 measurements (2012–2015).

The results are shown in Figure 13. The areas where the slope variation value is greater than 0.4 are 11–23 and 80–90 km along the railway, with the maximum value reaching 0.7‰. The maximum change in the regional settlement slope along the BTIR will reach 18‰ in its design life, which is less than the standard value of 20‰. These two sections are also two sections with a large difference in the settlement rate, as shown in Figure 4a. The high-speed railway subsidence control standards in China stipulate that the maximum allowable differential subsidence between two adjacent points within 20 m is 20 mm.

In Section 4.3, we identified the section with significant uneven deformation characteristics. Based on the TSX observation results, the differential settlement of the section was calculated at 20 m intervals. The maximum cumulative differential settlement of the CTS is 4.5 mm/20 m between 2010 and 2015, which is smaller than the standard value. However, if deviation from this development occurs, the difference in settlement will increase year by year and exceed the standard value.

The decrease in regional ground elevation and the uneven settlement change of the railway slope affect the track stability, and even threaten the safe operation of the high-speed railway. Land subsidence monitoring along the high-speed railway should be strengthened, groundwater exploitation along the line should be strictly controlled, and the track smoothness and pavement cracks should be regularly checked to ensure the safe operation of the high-speed railway.

## 6. Conclusions

In this study, we applied an advanced InSAR time-series technique, StaMPS, to two stacks of SAR data to investigate the subsidence along the BTIR. The combination of RADARSAT-2 and TSX data solves the contradiction between the width and resolution of SAR images in settlement monitoring along large-scale man-made linear features. RADARSAT-2 data with large coverage were used to obtain subsidence data along the whole railway. From the RADARSAT-2 investigation, we identified two severe subsidence areas. One of these severe subsidence areas is located in the CTS of the railway, and the other area is located in the WBC, with the maximum settlement rate reaching −71 and −20 mm/yr, respectively. Then, high-resolution SAR data from the TSX were used to reveal details of the CTS with InSAR analysis. With the TSX measurement results, we identified a significant uneven settlement region from 12 to 20 km along the railway and from 120 to 20 m on both sides of the CTS. Differential settlement changes the railway slope, which has negative effects on track stability. Fortunately, the maximum change due to regional settlement of the railway slope is within the safe range, according to the high-speed railway design standards. The maximum cumulative differential settlement of the CTS is 4.5 mm/20 m, which is also smaller than the standard value between 2010 and 2015. The analysis of the measured displacement and groundwater depth levels along the railway shows that the relationship between the groundwater and surface displacement levels varies at different locations. The results suggest that regional groundwater change is the main driving force of the development of uneven subsidence. Other triggering factors, such as dynamic loads, often have a certain influence on or contribution to differential subsidence.

Regional land subsidence and its differences will cause changes in the slope of high-speed railways, affecting the safe operation of the track. The InSAR technique provides a potential solution for consecutive health monitoring of railways with weekly data updates and a high accuracy. Combined with GIS spatial analysis, the Ex and En method can detect uneven settlement regions along the railway. We will consecutively monitor the health condition of the BTIR and detect changes at an early stage. Additionally, we will calculate the contribution rate of each influencing factor to subsidence and build a BTIR operational safety assessment model. It is very important to monitor the subsidence along railways throughout the whole life cycle, which is scientific and effective for the control of land subsidence along linear features and early warning of railway operation risks caused by subsidence.

## Figures and Tables

**Table 1:** Comparison of the mean subsidence rate between the RADARSAT-2 StaMPS and leveling data at the 34 benchmarks.

Benchmark No.	2012.9–2013.9 (mm/yr)			Benchmark No.	2012.9–2013.9 (mm/yr)		
	StaMPS	Leveling	Difference		StaMPS	Leveling	Difference
1	−21.0	−26.4	5.4	19	−14.0	−28.5	14.5
2	−10.0	−13.9	3.9	20	−10.0	−21.8	11.8
3	−38.0	−53.1	15.1	21	−13.0	−24.8	11.8
4	−61.0	−68.4	7.4	22	−35.0	−37.3	2.3
5	−12.0	−16.0	4.0	23	−30.0	−33.7	3.7
6	−32.0	−41.7	9.7	24	−18.0	−21.4	3.4
7	−49.0	−57.8	8.8	25	−15.0	−23.1	8.1
8	−16.0	−13.0	−3.0	26	−20.0	−24.1	4.1
9	−31.0	−38.0	7.0	27	−23.0	−25.7	2.7
10	−5.0	0.6	−5.6	28	−2.0	−17.3	15.3
11	−5.0	2.8	−7.8	29	−12.0	−29.2	17.2
12	−20.0	−20.9	0.9	30	−3.0	−18.5	15.5
13	−25.0	−28.2	3.2	31	−3.0	−18.0	15.0
14	−28.0	−30.3	2.3	32	−1.0	−16.1	15.1
15	−6.0	−3.5	−2.5	33	−41.0	−43.6	2.6
16	−5.0	−5.6	0.6	34	−5.0	−1.8	−3.2
17	−16.0	−14.0	−2.0	35	−4.0	−2.0	−2.0

**Table 2:** Comparison of the mean subsidence rate between the TSX StaMPS and leveling data at the 16 benchmarks.

Benchmark No.	2012.9–2013.9 (mm/yr)		
	StaMPS	Leveling	Difference
2	−15.0	−13.9	−1.1
3	−43.0	−53.1	10.1
4	−68.0	−68.4	0.4
5	−19.0	−16.0	−3.0
6	−38.0	−41.7	3.7
8	−16.0	−13.0	−3.3
10	−15.0	0.6	−15.6
11	−12.0	2.8	−14.8
12	−20.0	−20.9	0.9
15	−10.0	−3.5	−6.5
16	−10.0	−5.6	−4.4
17	−18.0	−14.0	−4.0
18	−42.0	−39.0	−3.0
20	−19.0	−21.8	2.8
34	−11.0	−1.8	−9.2
35	−11.0	−2.1	−9.0

**Table 3:** The information of wells.

Name	Elevation (m)	Monitoring Depth (m)	Underground Water Type
DFZ	30.10	70.60–92.00	confined
BLQ	25.30	48.00–94.60	confined
		157.80–238.40	
MJQ	27.23	56.00–100.00	confined
